# Quantification of Normal Cell Fraction and Copy Number Neutral LOH in Clinical Lung Cancer Samples Using SNP Array Data

**DOI:** 10.1371/journal.pone.0006057

**Published:** 2009-06-26

**Authors:** Hanna Göransson, Karolina Edlund, Maria Rydåker, Markus Rasmussen, Johan Winquist, Simon Ekman, Michael Bergqvist, Andrew Thomas, Mats Lambe, Richard Rosenquist, Lars Holmberg, Patrick Micke, Johan Botling, Anders Isaksson

**Affiliations:** 1 Department of Medical Sciences, Uppsala University, Uppsala, Sweden; 2 Department of Genetics and Pathology, Uppsala University, Uppsala, Sweden; 3 Department of Oncology, Uppsala University Hospital, Uppsala, Sweden; 4 AstraZeneca, Alderley Park, Macclesfield, United Kingdom; 5 Department of Medical Epidemiology and Biostatistics, Karolinska Institute, Stockholm, Sweden; 6 Regional Oncologic Centre, Uppsala University Hospital, Uppsala, Sweden; 7 Division of Cancer Studies, Medical School, King's College London, London, United Kingdom; 8 Department of Surgical Sciences, Uppsala University, Uppsala, Sweden; Deutsches Krebsforschungszentrum, Germany

## Abstract

**Background:**

Technologies based on DNA microarrays have the potential to provide detailed information on genomic aberrations in tumor cells. In practice a major obstacle for quantitative detection of aberrations is the heterogeneity of clinical tumor tissue. Since tumor tissue invariably contains genetically normal stromal cells, this may lead to a failure to detect aberrations in the tumor cells.

**Principal Finding:**

Using SNP array data from 44 non-small cell lung cancer samples we have developed a bioinformatic algorithm that accurately models the fractions of normal and tumor cells in clinical tumor samples. The proportion of normal cells in combination with SNP array data can be used to detect and quantify copy number neutral loss-of-heterozygosity (CNNLOH) in the tumor cells both in crude tumor tissue and in samples enriched for tumor cells by laser capture microdissection.

**Conclusion:**

Genome-wide quantitative analysis of CNNLOH using the CNNLOH Quantifier method can help to identify recurrent aberrations contributing to tumor development in clinical tumor samples. In addition, SNP-array based analysis of CNNLOH may become important for detection of aberrations that can be used for diagnostic and prognostic purposes.

## Introduction

**Figure 1:**
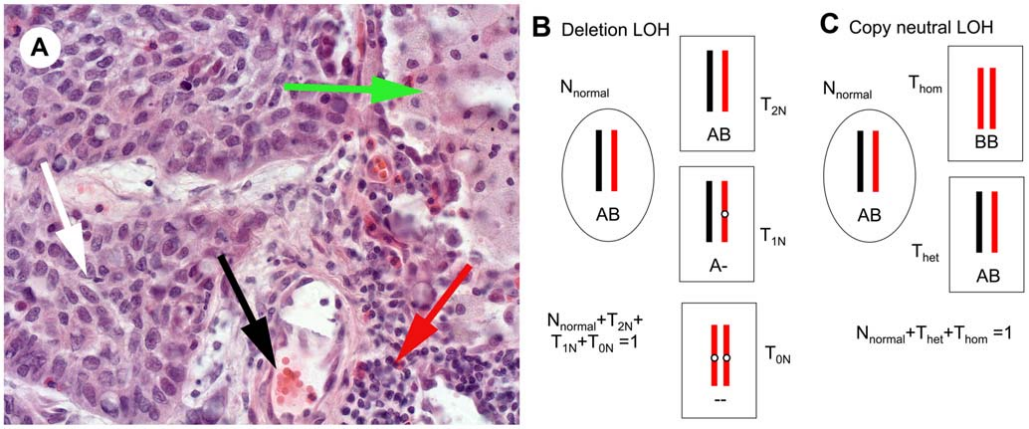
Tumor sample heterogeneity. A) Hematoxylin-eosin stained frozen section of a representative NSCLC case analysed in this study (original magnification 40×). The tumour sample is composed of a mixture of tumor cells white arrow, stroma with a blood vessel black arrow, inflammatory cells, i.e lymphocytes red arrow, and a remaining lung alveolus filled with macrophages green arrow. B) Deletion LOH. Normal cells are indicated by oval cell shape. The deleted region in the tumor cells (rectangular cell shape) is indicated by white circles. Schematic chromosome pairs with only informative markers A for the black and B for the red chromosme. Below each chromosome the genotype in the area of the deletion is indicated. Cells with different copy number genotypes are labelled N_normal_, T_2N_, T_1N_, and T_0N_. N indicates that the cell is normal and T that it sis a tumor cell. The subscripts for the tumor cells indicate the DNA content in the region with deletion. Since these are all the cell types in the sample the proportions sum up to one. C) Copy neutral LOH. In this case the tumor cells all have 2N DNA content. However, some tumor cells are homozygous (in this case BB) for the region of interest (T_hom_) and the other type is heterozygous AB (T_het_). The sum of the fractions of cells equals one.

Bioinformatic algorithms have been developed to use SNP array information to identify genomic aberrations such as DNA copy number changes and loss-of–heterozygosity (LOH), i.e. stretches of DNA with exclusively homozygous markers [1]–[8]. However, one major drawback of these methods is that genetic heterogeneity in tumor samples, caused by the mixture of cancer and stromal cells, is often not taken into account. As a consequence aberrations are often not detected in samples with a large proportion of genetically normal cells. This may partly explain why, despite the accumulation of large amounts of genomic data, the clinical impact of such analyses for diagnostic purposes is still small. Tumor tissue represents a mixture of tumor and non-tumor cells, i.e. inflammatory cells, stromal fibroblasts and cells of blood- and lymph vessels [9]. The fraction of normal cells often exceeds the fraction of tumor cells in patient samples stored in biobanks (Figure 1A). This sample heterogeneity severely affects copy number analysis. To the best of our knowledge there are no estimates on how the sensitivity of detection of genomic aberrations depends on the proportion of normal cells in clinical tumor samples. One reason may be the difficulty to estimate the tumor vs. normal cell ratio histologically by microscopy in heterogeneous tumor samples with varying proportions of normal cells in different parts of the sample. Moreover, there is a lack of consensus on how tumor cell content in a solid cancer should be assessed and annotated. Thus, the performance of the current tools for detection of genomic aberrations in clinical tumor samples is often uncertain.

A recently developed tool takes sample heterogeneity into account for identification of copy number states [10]. It is designed for studies with paired samples (tumor and normal). In practice, however, paired samples are often not available for larger patient cohorts.

In another study Nancarrow et al visualize the expected pattern of allele frequencies depending on varying proportions of normal cells in the tumor sample using simulations [11].

Another promising analytical tool, AsCNAR, is able to identify LOH even when one of two mixed cell lines is present only in a proportion of about 20% [12]. Recently Assie et al described an algorithm that take tumor heterogeneity into account in identifying genomic aberrations in samples with 40–75% of tumor cells [13].

Studies suggest that copy number neutral LOH can be a mechanism for inactivation of tumor suppressor genes [14]. Several studies and our own data suggest that CNNLOH is more common than previously thought [15], [16]. Taken together this suggests that CNNLOH may be important in determining certain cancer phenotypes. To analyze CNNLOH on a genome-wide scale in the tumor cells in heterogeneous samples we focused on 1) developing an algorithm to quantify the proportion of normal cells in the sample and 2) to quantify CNNLOH throughout the genome in the tumor cells. Such quantitative analysis has the potential to become an important tool for molecular cancer diagnostics.

## Results

### A strategy for quantification of CNNLOH in heterogeneous tumor samples

To quantitate CNNLOH in heterogeneous tumor samples the allele-specific signal contribution from different types of cells need to be estimated. Figure 1 illustrates a typical mixture of cells in frozen sections of a non-small cell lung cancer (NSCLC) tumor sample and provides a schematic representation of the different of types of cells and genotypes that could be present in the event of a genomic deletion or CNNLOH. Other genomic aberrations, including those giving rise to higher ploidy aberrations, may also occur at the same locus as the deletion or CNNLOH, further complicating the picture. However, the likelihood of such events can be expected to be low and in this study they have been assumed to be negligible in comparison to the effects of deletions and CNNLOH.

The fraction of normal cells can for some types of tumors be measured in a straightforward manner. In hematological tumors the fraction of normal cells can be measured using flow cytometry employing informative surface markers. However, single cell suspensions for flow cytometry are difficult to obtain from solid tumors. Alternatively, automated or manual means of identifying normal cells by counting them *in situ* based on molecular markers or morphology may be used. These methods require advanced imaging techniques or time consuming work for a trained histopathologist. In this paper we use the signal intensities from the two SNP alleles to estimate the fraction of normal cells and use that information to estimate the proportion of tumor cells with CNNLOH.

### Quantification of the proportion of normal cells from SNP genotyping array data

**Figure 2:**
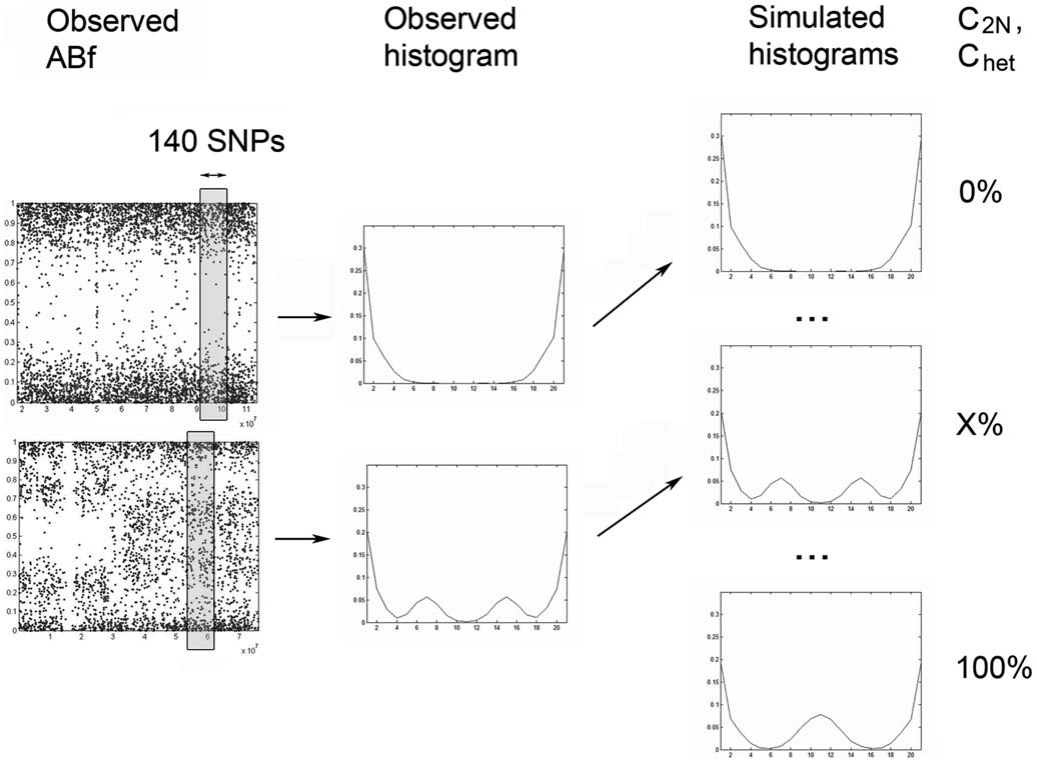
Estimation of the proportion of heterozygous cells. Two examples of observed ABf values along chromosomes. Windows of at least 140 consequtive SNP markers are used to gather allele-specific information along the chromosome. The ABf values in each window are illustrated as an observed histogram. In the next step the observed histogram is compared to hundreds of simulated histograms where the fraction of heterozygous cells has been varied. The simulated histogram with the shortest Euclidean distance to the observed histogram identifies the most likely proportion of heterozygous cells in the sample (X%). The comparison of histograms is used both for estimation of the proportion of diploid cells (C_2N_) in regions where tumor cells have deletions and the proportion of heterozygous cells (C_het_) in genomic regions with 2N tumor cells. C_2N_ and C_het_ are subsequently use for estimation of the fraction of normal cells and quantification of CNNLOH.

In samples where the proportion of normal cells is difficult to obtain by other means, we set out to estimate the fraction of normal cells from SNP data only. As shown in Fig. 1B the fraction of cells with 2N DNA (C_2N_) in regions with deletions is the sum of the normal cells (N_normal_) and the tumor cells with 2N DNA (T_2N_). The basis for the CNNLOH Quantifier method is to use the allele-specific signals A and B for each locus to obtain the experimental Allele B frequency (ABf), as a normalized ratio of B/(A+B) see METHODS. A derivation and a graphical illustration are available elsewhere [17]. The ABfs of heterozygous informative markers in complex tumor samples depend on copy number and cellular composition of the sample. In a first step we set out to identify the proportion of C_2N_ cells by comparing the experimental ABfs in a window of consecutive SNPs in regions with mono-allelic deletions with simulated data. The simulations take factors into account such as average heterozygosity, tumor cell copy number, experimental variation and the composition of diploid cells and tumor cells. Figure 2 illustrates how histograms of observed ABfs are compared to simulated histograms with varying fractions of C_2N_ using the Euclidean distance. The histogram with the smallest distance to the observed histogram identifies the corresponding C_2N_ (see Fig. 2 and METHODS).

N_normal_ is not obtained directly from the estimate of C_2N_. However, since the normal cells can be expected to have two of each autosome their contribution to the ABf is expected to be equally large for all autosomes, while the additional contribution of T_2N_ cells may vary with the tumor heterogeneity for each chromosome. Thus, in samples where copy number loss is detected on several chromosomes the lowest estimate of C_2N_ for a chromosome has the smallest contribution from 2N tumor cells. In many cases where the deletion has caused a proliferation advantage the contribution to the smallest C_2N_ from 2N tumor cells will with time be close to zero. Therefore in cases where information from several chromosomes is available it may be justified to estimate N_normal_ as the smallest C_2N_.

### Validation of SNP array based estimates by comparison to manual counting of normal cells

We applied the method to 60 non-small cell lung cancer samples (see METHOD). Forty-four out of sixty samples (73%) met the arbitrary criteria that at least two regions on two different chromosomes varied no more than 5% in their C_2N_ estimates and that could be used to provide an estimate of N_normal_ (see METHODS). The criteria have been set to take into account the possible effect of constitutive allelic patterns resembling CNNLOH. Most of the 16 cases for which no estimate could be obtained did not have two deletions.

In order to validate the estimates of the normal cell fraction based on the SNP data, a random subset of the 60 NSCLC samples were selected for careful and extensive microscopic counting of normal and tumor cells in frozen sections (see METHODS). Seven out of these cases overlapped with the 44 cases where N_normal_ could be estimated, Table 1 shows the estimates of the fraction of normal cells obtained using SNP array data and manual counting based on morphology for these samples. There is good agreement between the results obtained by the two methods, which indicates that the SNP based method provides accurate information on the fraction of normal cells in tumor samples.

**Table 1:** Estimation of the fraction of normal cells.

Sample	Fraction of normal cells, SNP-based	Fraction of normal cells, counting
367A	0.23	0.29
347A	0.58	0.50
319A	0.23	0.23
234A	0.17	0.20
189A	0.45	0.48
165C	0.42	0.38
39A	0.52	0.55

Comparison between estimates of the fraction of normal cells in Non-Small Cell Lung Cancer samples using either manual light microscope counting or the SNP array based method. (see METHODS).

### Quantitation of copy number neutral LOH in lung cancer samples

**Figure 3:**
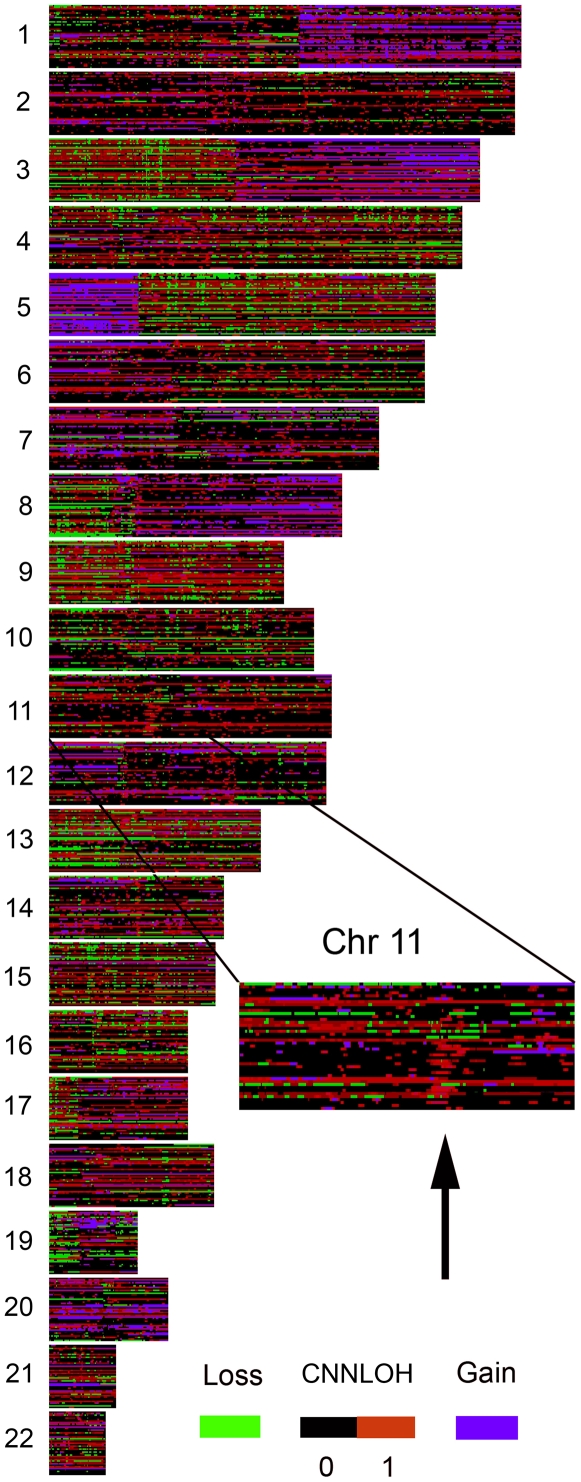
Genome-wide quantitation of copy number neutral tumor LOH. A) Quantitative information on tumor LOH ranging from black 0% to red 100% for the 22 autosomes for 43 non-small cell lung cancer samples each representing one row. Deletions are indicated by green and amplifications in blue. Regions where more than 10% of the samples in a normal reference set had CNNLOH higher than 0.5 was removed from the plot (see METHODS). Black arrow indicates an example of a region on chromosome 11 with a higher frequency of copy number neutral LOH (52%) than the maximum frequency of 10% in the normal reference set.

It is necessary to quantify the fraction of normal cells present in a tumor sample before information from SNP array analysis can be used to estimate the fraction of tumor cells with 2N DNA that has LOH, i.e. CNNLOH. CNNLOH  =  T_hom_/(T_hom_+T_het_). (see Fig. 1B). In this case it is the heterozygous tumor cells T_het_ that together with the normal cells will modify the Allele B frequency of the homozygous tumor cells with LOH. For CNNLOH the Allele B frequency of the informative markers depends on the heterozygous cells C_het_. Simulated histograms taking varying fractions of heterozygous cells into account were compared to histograms based on ABf values from a moving window with a fixed number of markers (see Fig. 2 and METHODS). The simulated histogram most similar to the observed histogram, identified the corresponding fraction of heterozygous cells, C_het_. If the fraction of normal cells N_normal_ is known, CNNLOH can be calculated, since 1 =  N_normal_ + T_hom_+T_het_. To demonstrate the value of CNNLOH Quantifier method we applied it to a set of NSCLC samples (see METHODS). Genome-wide quantitative measurements of CNNLOH are shown in Fig. 3. It can be noted that there are recurring regions with high degrees of copy number neutral LOH. One example on chromosome 11 is shown in Fig. 3. Future work will be focused on elucidating the importance of copy number neutral LOH in such regions for tumor development. For comparison CNNLOH was also analyzed in 60 normal reference samples (see Fig. S1).

### Quantification of CNNLOH – comparison between FISH and SNP data

In order to study the accuracy of the quantification of CNNLOH we wanted to compare the results to those obtained with an independent method. Gunnarsson et al have measured the presence of small deletions on 13q14 in tumor cell preparations from patients with chronic lymphocytic leukemia (CLL) using fluorescence in situ hybridization (FISH) [18]. In two cases tumor cells had acquired two copies of chromosome 13 with an internal 13q14 deletion. Thus, in these cases the proportion of CLL cells with zero and one FISH signal represent the homozygous and heterozygous tumor cells respectively. An estimate of the CNNLOH on chromosome 13 outside of the deleted region on 13q14 can be obtained as the proportion of cells with zero signal on 13q14 divided by the sum of the proportions with zero and one signal. Due to too few regions with deletions the proportion of normal cells could no be obtained from the SNP data. Instead flow cytometry data from Gunnarsson et al 2008 provided this information. Thus, the proportion of normal cells from flow cytometry and SNP array data for the two samples was used to quantify CNNLOH. Table 2 shows the estimates of CNNLOH obtained by the two methods. It can be noted that the estimates only differ by 4 and 6% in the two samples, which indicates that the measurement of CNNLOH is accurate.

**Table 2:** Validation of CNNLOH estimates using FISH.

Sample	SNP-array	FISH
6	44%	38%
7	84%	88%

CNNLOH estimates just outside of the chromosome 13q14 region in two CLL samples based on analysis of SNP data compared with CNNLOH estimates obtained from FISH analysis on chromosome 13q14 in Gunnarsson et al 2008. Due to too few regions with deletions in these samples SNP data could not be used to quantify the fraction of normal cells. Instead flow cytometry data from Gunnarsson et al 2008 was used to obtain the fraction of normal cells. This information together with the SNP array data was used to quantify CNNLOH.

### Quantification of CNNLOH is robust to variations in sample purity

**Figure 4:**
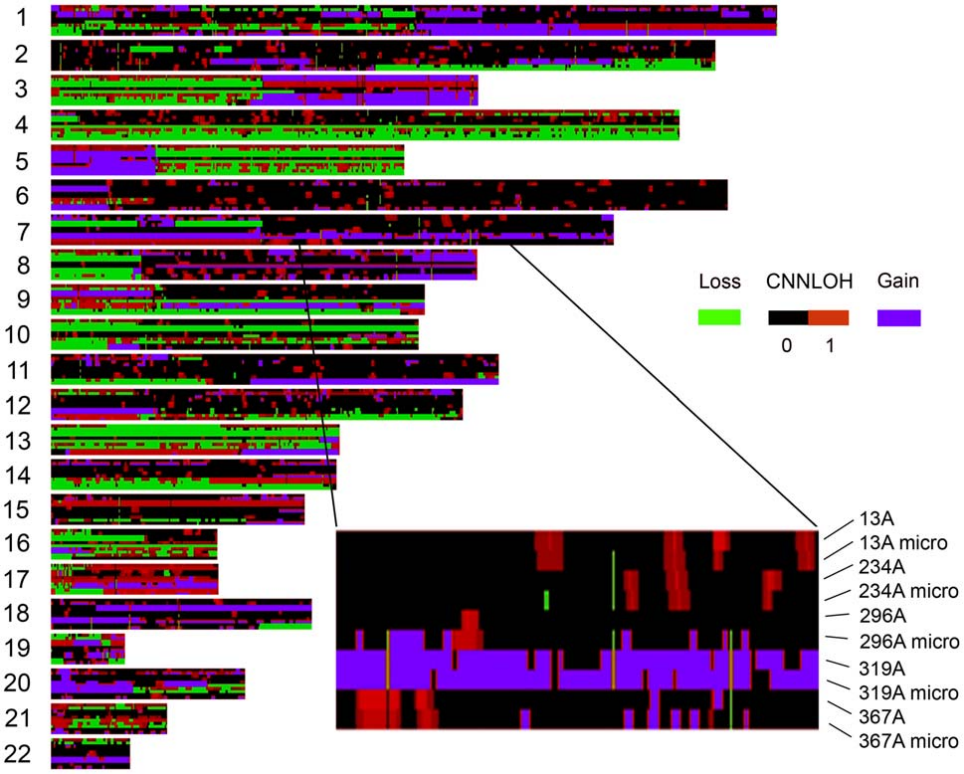
Tumor LOH analysis is robust to variations in normal cell content in crude tumor samples and enriched tumor preparations from the same cases. Five tumor samples 13A, 234A, 296A, 319A and 367A were analyzed for tumor LOH using both DNA from fresh frozen sections of the whole tumor and from laser microdissected portions of the tumor sections with enriched tumor content. The degree of tumor LOH is indicated for 200 kb chromosmal segments in color ranging from black (0%) to dark red (100%) along the 22 autosomes. Segments with copy number aberrations are indicated with deletions (green) and amplifications (blue). Note in an enlarged section of chromosome 7 that the CNNLOH measurements are approxiamately equal in the pairs of whole and microdissected tumor samples.

An important requirement for the quantification of CNNLOH is that the method should be robust and not influenced by the fraction of normal cells in the sample. In order to test the performance of the algorithm we varied the fraction of normal cells in a tumor sample by enriching for tumor cells. In five cases 6 000–10 000 tumor cells were selected using laser microdissection (see METHODS). Standard Affymetrix SNP array analysis was performed on DNA from these tumor-enriched preparations. The fraction of normal cells and tumor LOH for copy number neutral regions was quantified and the results were compared to the results from crude tumor samples (Fig. 4). The measurements of CNNLOH in the crude samples appear to be highly consistent with those in the microdissected samples. In order to quantify the performance of CNNLOH detection we chose to count the number of chromosomal segments in Fig. 4 that had tumor LOH higher than an arbitrarily set threshold, in this case 0.5 in both samples. This procedure provides a rough estimate of the ability to detect CNNLOH. Table 3 shows the ratio of number of segments that were detected in the microdissected sample compared to the whole sample. The ratio is close to one for all samples indicating that approximately the same numbers of segments are detected irrespective of fraction of normal cells that varied between 1% and 26%. It could be argued that the fraction of normal cells is so low that it is difficult to observe any difference between the pairs of samples. However, studying the robustness of copy number detection it can be noted that in three pairs of samples (13A, 296A and 319A) there was a dramatic reduction (up to 122-fold) in the number of segments detected as bearing copy number aberrations in the whole sample (see Table 3.). In summary, the analysis of CNNLOH appears to be robust, while copy number detection may be highly influenced even by a proportion of normal cells in the range of 1–26%, which is modest for many types of clinical tumor specimens.

**Table 3:** Quantitation of copy number aberrations and CNNLOH in samples with varying proportions of normal cells.

*Sample*	Segments with CN loss	Segments with CN gain	Segments with CNNLOH>0.5
13A, N = 0.26	264	590	900
13A micro, N = 0.11	1349	1502	1226
***13A micro/13A***	**5.0**	**2.6**	**1.4**
234A, N = 0.20	1568	988	831
234A micro, N = 0.14	1581	953	808
***234A micro/234A***	**1.0**	**1.0**	**1.0**
296, N = 0.36	16	226	302
296 micro, N = 0.02	1958	887	505
**296 micro/296A**	**122**	**3.9**	**1.6**
319, N = 0.23	1036	1823	1274
319 micro, N = 0.01	2221	2116	1269
**319 micro/319A**	**2.1**	**1.2**	**1.0**
367, N = 0.23	2446	1324	873
337 micro, N = 0.01	2649	2026	593
**367 micro/367A**	**1.1**	**1.5**	**0.7**

The number of genomic segments with predicted copy number gain or loss is indicated for each sample. The number of segments with CNNLOH larger than 0.5 and no copy aberrations detected in either analysis are also shown. The ratio of the number of aberrations in the microdissected and whole samples are indicated. For the samples with large differences in detection of segments with copy number differences, such as 13A, 296A and 319A, the corresponding detection of CNNLOH appears to be more robust. The proportion of normal cells for each sample is obtained either from manual counting (whole samples) or by the method presented here (microdissected samples).

### Performance of CNNLOH detection on simulated data of mixtures of normal and tumor cells

**Figure 5:**
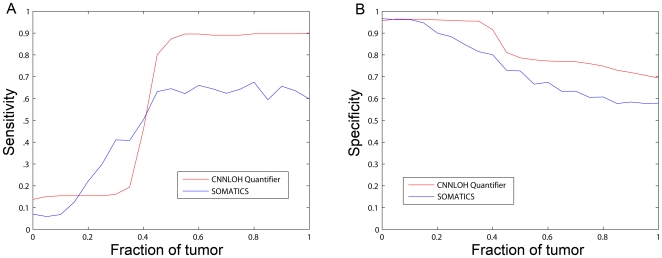
Performance of tumor-only analysis using CNNLOH Quantifier and SOMATICS on simulated data corresponding to virtual mixtures of normal and tumor cells. Data corresponding to varying fractions of normal and tumor cells was simulated using data from normal cells and microdissected tumor cells from the same lung cancer tumor. The performance of the two algorithms was evaluated as sensitivity (A) and specificity (B) compared to CNNLOH detected by dChip in a paired analysis of SNP array data of the normal and tumor cells.

The CNNLOH Quantifier method presented here can identify CNNLOH and quantify the fraction of tumor cells that has CNNLOH. The method appears to be robust to variations in the tumor cell content in clinical specimens. However, it would also be interesting to compare its performance to other methods. To this end SNP array data was collected from tumor cells in a microdissected lung cancer sample and from lung cancer tissue outside of the tumor from the same patient. Based on this information we simulated data from mixtures of normal and tumor cells. In order to measure performance in terms of sensitivity and specificity we used LOH detected by paired analysis of the tumor sample and its normal control using dChip in copy number 2 regions as the gold standard that defined CNNLOH. We wanted to compare the CNNLOH Quantifier method to other methods also using allele-specific information. These methods have been shown to outperform genotype-based methods [13]. AsCNAR is one such method but the presently available implementation is not flexible enough to use on this type of simulated data. On the other hand the SOMATICS method was designed for data from the Illumina SNP platform, but could be adapted to analyze simulated data based on data from the Affymetrix platform. Therefore we chose to compare performance of the CNNLOH Quantifier method with SOMATICS (see METHODS for details). Sensitivity and specificity of the methods for varying mixtures of normal and tumor cells are shown in figure 5AB. It can be noted that CNNLOH Quantifier has a sharp increase in sensitivity, above 40% tumor cells, that is due to the threshold of CNNLOH calling that corresponds to a fraction of 35% tumor cells. CNNLOH Quantifier has a higher sensitivity of about 90% compared to 60% for SOMATICS for fractions of tumor larger than about 50% (see Fig. 5A). Specificity is generally higher for CNNLOH Quantifier compared to SOMATICS (see Fig. 5B). The sensitivity of SOMATICS was lower than what has been previously reported for data based on SNP array data from the Illumina platform [13]. We hypothesized that the SOMATICS algorithm performs differently on data generated by the two platforms. In Gunnarsson et al the same DNA preparations from CLL tumors were analyzed on both 250K arrays from Affymetrix and 317K arrays from Illumina [18]. We analyzed both data sets from 9 CLL tumors with SOMATICS and found that the algorithm identified more CNNLOH using Affymetrix data. The ratios of the length of the detected CNNLOH regions with Affymetrix data compared to Illumina data range from 1.9 to 36 (see Table S1). These additional regions of CNNLOH identified using the Affymetrix data may to a large extent be false positives due to the larger experimental noise. Such a large number of false positives would be consistent with the low sensitivity of SOMATICS in detection of CNNLOH in the simulated data based on Affymetrix data (see Fig. 5A).

## Methods

### Ethics statement

This study was conducted according to the principles expressed in the Declaration of Helsinki and was approved by the regional ethical review board in Uppsala (reference number 2006/325). The need to obtain individual consent from each patient was waived by the ethical review board, since most of the patients of the study population (>90%) were deceased at the start of the project, the research results did not imply any medical risks, and the results could not alter the information to the patients, their families or change management. The procedure is in full agreement with the Swedish Ethical Review Act.

### Tumor samples, histological estimation of tumor cell content and DNA preparation

Fresh frozen human NSCLCs were obtained from the Uppsala Fresh Tissue Biobank and used in accordance with the Swedish biobank legislation. The tumor specimens emanated from patients from a cohort defined by that they were registered in the Uppsala/Örebro lung cancer registry as having NSCLC, had a frozen tissue sample in the tissue bank and were diagnosed while alive. Case status was verified by histopathological review and study of medical records. Hematoxylin-eosin stained cryosections (4 µm) were prepared from the frozen OCT-embedded tumor tissue blocks and reviewed microscopically by a surgical pathologist. Sixty cases with an estimated tumor cell content over 50% were included in the study. For a subset of these samples (Table 1) we performed a careful manual counting of tumor and normal cells by light microscopy using grids in high power magnification fields (hpf). At least 2000 cells were counted in 5–10 different areas of the frozen section depending on the size and heterogeneity of the tumor sample. For each sample genomic DNA was extracted from 5–10 frozen tissue sections (10 µm) using the QIAamp DNA Mini Kit (Qiagen) according to the manufacturer's protocol.

### Laser Capture Microdissecion of lung cancer samples

Microdissection was performed as previously described with minor modification [19]. From 5 lung cancer samples, 12 µm thick cryosections were prepared, transferred to PALM membrane-coated glass slides, and stored at −80°C. Immediately prior to microdissection, sections were thawed and stained with hematoxylin for 2 minutes followed by fixation in a zinc fixative for 1 minute and dehydration for 1 minute in 70% and 95% ethanol respectively. Utilizing the PALM Laser-MicroBeam System, selected tumor areas containing 6000–10000 cells were microdissected and transferred by means of a laser puls to 15 µl DNA extraction buffer ATL (Qiagen) in the cap of a microfuge tube. DNA extraction was then performed using the QIAamp DNA Micro Kit according to the manufacturer's protocol (Qiagen).

### Analysis on Affymetrix 250K SNP arrays

Array experiments were performed according to the standard protocols for Affymetrix GeneChip® Mapping 250K arrays (Gene Chip Mapping 500K Assay Manual (P/N 701930 Rev2.), Affymetrix Inc., Santa Clara, CA). Briefly, total genomic DNA was digested with a restriction enzyme (*Nsp1*), ligated to an appropriate adapter for the enzyme, and subjected to PCR amplification using a single primer. After digestion with DNase I, the PCR products were labeled with a biotinylated nucleotide analogue using terminal deoxynucleotidyl transferase and hybridized to the microarray. Hybridized probes were captured by streptavidin-phycoerythrin conjugates and finally the arrays were scanned. Quality control QC, genotype calling and copy number analyses were made in the Affymetrix GeneChip® Genotyping Analysis Software (GTYPE) 4.1. The Dynamic Model (DM) algorithm was used to perform single sample QC. The QC specification for 250K is a Call Rate >93% using the algorithm defaults. Subsequent copy number analysis was performed using a Hidden Markov Model available in the Copy Number Analysis Tool (CNAT) 4.0.1 with the following parameter settings: Transition decay 5 Mb, Median normalization and 0.3 Mb smoothing factor. The reference set used consisted of 96 CEU samples from the HapMap project (www.hapmap.org/downloads/raw_data/affy500k/).

### Quantification of the fraction of normal cells

As a first step genomic regions inferred to contain mono-allelic deletions were identified using the Affymetrix Software CNA 4.0.1 as described above. In order to estimate the fraction of cells with 2N genomic content, C_2N_, allele-specific information was used. The Affymetrix SNP raw data was normalized in the software dChipSNP using the model-based expression method and a background subtraction method that uses mismatch probes (PM/MM difference) [6]. The normalized signals were then used to calculate allelic intensity ratios R_i_ for each SNP (R_i_ = B/(A+B)). To take into account that the same number of A and B alleles may produce different signals in the assay the allelic ratios were normalized to allele B frequencies (ABf) for a particular SNP locus in a given sample by a linear interpolation of the known allele frequencies for the three genotypes (0, 0.5 and 1.0), as derived and graphically illustrated in Peiffer *et al* 2006 [17]. In short the ABf for a given SNP *i* was calculated as follows:
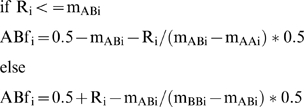
where R_i_ is the allelic intensity ratio and m_AA,AB,BB_ are the mean allelic intensity ratios for a reference population, for the particular SNP. The reference set was the same as described for copy number analysis above. Only the SNPs where an AB call was present in the reference population were used. For the SNPs where no homozygous calls were available, the mean AA or BB signal for all SNPs in the samples in the reference population was used instead.

A histogram of the allele-specific Allele B frequency information for the markers on each autosome with copy number loss was computed. The observed histogram was compared to simulated histograms for varying fractions of C_2N_. The C_2N_ of the most similar histogram is the one most likely to have given rise to the observed data (see Fig. 2). The simulations describe the variation in ABf due to variation in the signals from A and B alleles by drawing A and B signals from normal distributions estimated from regions with copy number 2. The mean and variance for the distributions were (0, 0.015) for allele A and (0.995, 0.015) for allele B. The average degree of heterozygosity was estimated from copy number 2 regions to 36.7%. Simulated ABf values were calculated as the sum of simulated Allele B signals divided by the sum of Allele A and B signals from cells composing the virtual sample. For example when simulating samples with higher fractions of 2N cells, the proportion of simulated cells with both A and B signals is also increased. To maintain the average degree of heterozygosity and to obtain stable histograms they were based on the ABf values from the uneven number of 14 848 simulated SNP loci (see more detailed description of choice of settings in Text S1). Choosing another sufficiently large number of simulated loci would produce very similar histograms. The histograms were normalized to unit area to represent the expected pattern for each of the 200 steps that varied the fraction of C_2N_ cells between 0 and 67%. The smallest Euclidean distance between the observed histogram and the histograms with varying C_2N_ identified the fraction most likely to have given rise to the observed Allele B frequency pattern. Due to the distribution of the ABf values of the informative markers around 0.5 we chose to use only bins 5 to 16 to increase the weight of their information. To obtain reasonably well populated and robust histograms, we chose a minimum of 140 markers/chromosome for estimation of C_2N_. The smallest number that will give acceptable performance will depend on the experimental noise of the data and may vary between data sets. At least two regions on two different chromosomes that varied less than 5% in their C_2N_ estimates were required for min(C_2N_) to be used as an estimate of N_normal_ (see motivation of choice of settings in Text S1).

### Quantitation of copy number neutral tumor LOH in tumor-only samples

In the case of CNNLOH it is the fraction of heterozygous cells C_het_ that modify the Allele B frequency of the homozygous tumor cells with LOH. C_het_ = T_het_+N_normal_. Histograms were simulated in the same way as for as above except taking into account the 2N tumor DNA content and varying C_het_ between 0 and 91% in 1000 steps. It can be noted that ABf of a tumor sample containing 2N homozygous tumor cells will be less affected by heterozygous cells than a sample with 1N tumor cells. These histograms were compared to normalized histograms based on 2N regions with 140 consecutive markers scanning the diploid regions. The smaller the window size the larger the risk for false positives. The performance of the algorithm will also be dependent on the experimental noise in the data set. We choose a window size of 140 markers to obtain reasonable resolution and performance on our data set. The simulated histogram most similar to a particular observed histogram identified the corresponding fraction of heterozygous cells, C_het_. If the fraction of normal cells N_normal_ is known, T_het_ = C_het_−N_normal_ and T_hom_ = 1−N_normal_−T_het_. Thus, we can estimate the fraction of tumor cells with LOH, which is defined as CNNLOH = T_hom_/(T_hom_+T_het_). In genomic regions with local stretches of homozygous SNP genotype calls in the normal cells the assumption of average homozygosity is violated and a non-tumor specific CNNLOH signal is detected. In order to avoid these cases genomic regions with a CNNLOH score higher than 0.5 in 10% or more of the samples of a normal reference set is removed from the analysis. This is a threshold similar to what has previously been used [20]. The reference set used was 60 CEU samples from the HapMap project. A plot of CNNLOH in the 60 reference samples with the regions above the threshold removed is shown in Figure S1.

### Visualization of CNN LOH data

Regions with CNNLOH estimates are mapped to virtual 200 kb genomic regions for each chromosome in each sample. In order to illustrate the tumor LOH information from several samples the virtual probes with tumor LOH information from at least one sample are visualized using the DIGMAP software [21]. The virtual probes without LOH information are assigned the average value of neighboring probes with information. Copy number information for the virtual probes is overlaid the tumor LOH information.

### Simulations and analyis of SNP array data from mixtures of normal cells and tumor cells

SNP array data including Log2- and Allele B frequency values from the microdissected lung cancer sample 13A and the corresponding normal genomic DNA called 13C from the surrounding normal lung tissue was collected. The Allele B frequency data for virtual mixtures of these two samples (from 0% to 100% in 5% steps) was simulated by linear interpolation for each marker. Log2-values and copy number information from the microdissected tumor sample was used for all virtual samples. CNNLOH Quantifier and SOMATICS was used to analyze the simulated data. The results were compared to a gold standard which was the LOH detected in copy number 2 regions in a paired analysis of the tumor sample 13A and the paired control 13C using dChip. All markers in copy number 2 regions with an ABf-pattern corresponding to 35% homozygous cells or higher were considered as CNNLOH for the CNNLOH Quantifier algorithm. Markers detected as CNNLOH in more than 10% of a set of 60 reference samples from the HapMap project were regarded as false positives and removed from the analysis. Sensitivity and specificity of detection was measured for SOMATICS and CNNLOH Quantifier.

### Data and software

All microarray data reported here is described in accordance with MIAME guidelines and has been made publicly available through GEO with the accession number GSE16092. Computer code written in MATLAB is available from the authors upon request.

## Discussion

### Identification of informative SNPs

A key issue in detection of LOH and quantification of genomic aberrations in tumor samples is to identify the informative SNPs. The informative SNPs are those that are heterozygous in the normal cells of the patient. These markers can loose their heterozygosity and their allele frequency is dependent on the relative proportions of heterozygous normal and tumor cells, and tumor cells that have undergone LOH. In the case of paired analysis, a normal sample is available and the informative SNPs are those that are heterozygous in this sample. It is more difficult to identify the informative SNPs in a tumor-only analysis when the genotype of the normal sample is unknown.

One recently developed tool, AsCNAR, identifies the informative SNPs as those that are called heterozygous in the tumor sample [12]. This is a simple and efficient strategy in the common cases where there is a significant proportion of normal cells in the tumor sample. Another tool SOMATICs identifies informative SNPs based on statistical considerations. However, both of these tools have difficulty analyzing tumor samples with a low degree of heterogeneity, when the informative SNPs are difficult to distinguish from the uninformative ones. In contrast, CNNLOH Quantifier does not suffer from the same limitations, since it does not rely on identification of informative SNPs. Instead it uses a fixed average heterozygosity rate estimated from samples in a reference population. The risk that the fixed heterozygosity rate is not appropriate for every studied region in a particular sample appears to be small, since few regions are identified as having LOH in our reference population of normal samples. However, for all tumor-only methods including CNNLOH Quantifier it is difficult to exclude CNNLOH signals due to regions with homozygous markers in the constitutive DNA in pure tumor samples. CNNLOH Quantifier handles this issue by removing regions with more than 10% of the reference samples exceeding a 35% or 50% CNNLOH threshold from further analysis. However, even with such a threshold, CNNLOH signals due to signals from constitutive DNA may occur. Therefore we suggest validating CNNLOH findings in constitutive DNA when available. A possible improvement to the method described here could be to use a variable heterozygosity rate based on a moving average from the reference data set, but this has not been investigated further.

### Copy number information is required to interpret allele frequency information

Another fundamental feature of the algorithm described here is the need to have access to correct copy number information because it determines how much the SNP signal information is going to be affected by genetically normal cells. The Allele B frequency is affected more in regions with copy number one than with copy number 2 because the alleles from the normal cells constitute a larger proportion of all alleles. When estimating tumor heterogeneity using CNNLOH Quantifier an implicit assumption is made that there is only one type of genomic aberration at each locus. Although an important theoretical limitation, potential additional genomic aberrations appear to be small in magnitude or rare events in practice, at least in the tumors we studied, since the validation experiments indicate good accuracy of the method. One explanation is that many genomic aberrations provide a proliferation advantage. Thus, the cells containing these aberrations will become more frequent during tumor development than cells with other aberrations at the same locus not promoting proliferation or doing so to a lower extent.

The sensitivity to variation in normal cell content exhibited by current algorithms for detection of copy number aberrations in tumor cells, such as CNAT 4.0.1 used here, presents a more serious problem (see Fig. 4 and Table 3). Failure to adequately detect copy number aberrations provides inaccurate input data for the quantification of normal cells and CNNLOH. Undetected deletions will reduce the available information on which to base the quantification of normal cells on and may preclude determining the fraction of normal cells in some samples. For quantitation of CNNLOH undetected copy number 1 regions will be analyzed as copy number 2 regions and will therefore receive exaggerated estimates of CNNLOH. However, the deletions that are detected appear to be correct, since the estimates of normal cell content are in agreement with those based on microscopic cell count.

The evaluation of the performance of the CNNLOH Quantifier method indicates that it has a higher sensitivity than SOMATICS at least on data generated using Affymetrix SNP arrays (see Fig. 5A). The difference in performance of the SOMATICS algorithm depending on the data source may be explained by previously shown differences in experimental noise between data generated on the two platforms [18]. The SOMATICS algorithm identifies informative SNPs by statistical methods which becomes more difficult in the more noisy Affymetrix data. The CNNLOH Quantifier algorithm does not identify each informative SNP but studies the pattern of a group of consecutive SNPs and thus is more robust to experimental noise. The downside of studying a group of SNPs is that the method sometimes identifies too many markers as CNNLOH, which affects specificity negatively (see Fig. 5B).

### Summary

In this study we provide a novel algorithm, CNNLOH Quantifier, for genome-wide quantification of CNNLOH from SNP array data. We demonstrate that the fraction of normal cells in tumor samples, as well as copy number neutral LOH, can be accurately estimated in tumor cells. Since the use of SNP array data provides genome-wide information, it will now be possible to quantify common and rare events of CNNLOH in tumor cells from samples containing normal cells. Our algorithm may also be applied to screen for biomarkers that may be used for early detection of cancer. Additionally, this tool can be used to monitor how rapidly cells with a particular genomic aberration increase in a population of tumor cells during tumorigenesis. Given the complex nature of clinical tumor biobank material that forms the basis for translational cancer research, the development of bioinformatic tools that can process data from heterogeneous tumor samples with varying fractions of normal cells is of great importance.

## Supporting Information

Figure S1 Quantification of CNNLOH in the normal reference samples Quantitation of copy number neutral LOH in the 60 normal reference samples. Regions with CNNLOH above 0.5 in more than 10% of the samples have been removed. Note that allelic patterns of CNNLOH are present in several regions in individual samples. Thus, frequently recurring CNNLOH in tumor cells can be identified, while it is difficult to identify an individual tumor-specific CNNLOH event in an individual tumor sample.
(1.30 MB TIF)

Table S1 Size of the regions detected as CNNLOH using SOMATICS on Affymetrix and Illumina data from 9 Chronic Lymphocytic Leukemia samples described in Gunnarsson et al [18]. Ratio of the length of regions detected on Affymterix and Illimina data. Note that SOMATICS detects more CNNLOH using Affymetrix data than with Illumina data.
(0.03 MB DOC)

Text S1 Choice of settings.
(0.03 MB DOC)
