# Supplementary figures and images for: Quantification of Normal Cell Fraction and Copy Number Neutral LOH in Clinical Lung Cancer Samples Using SNP Array Data
Source: PLoS One. 2009 Jun 26;4(6):e6057. doi: 10.1371/journal.pone.0006057 (PMC2699026; doi:10.1371/journal.pone.0006057)

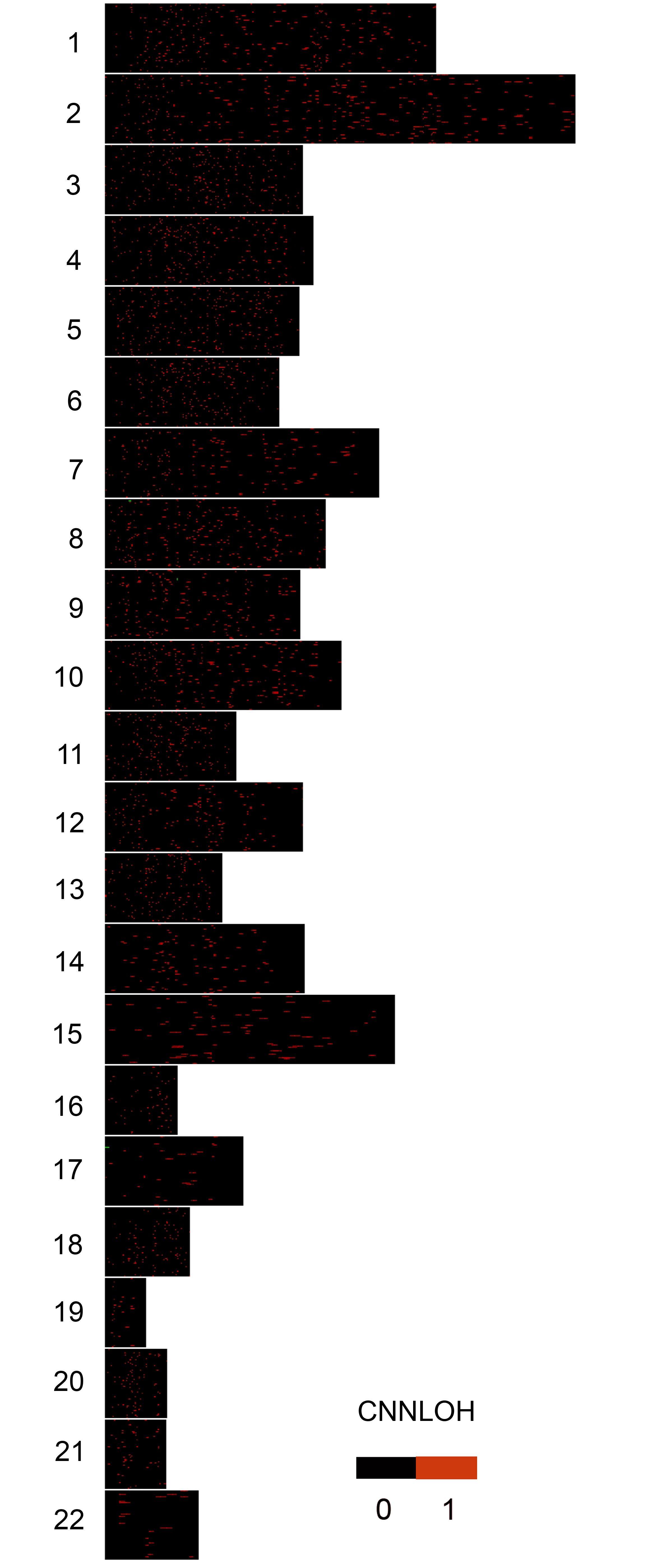

Supplement: Figure S1 — Quantification of CNNLOH in the normal reference samples Quantitation of copy number neutral LOH in the 60 normal reference samples. Regions with CNNLOH above 0.5 in more than 10% of the samples have been removed. Note that allelic patterns of CNNLOH are present in several regions in individual samples. Thus, frequently recurring CNNLOH in tumor cells can be identified, while it is difficult to identify an individual tumor-specific CNNLOH event in an individual tumor sample. (1.30 MB TIF) [file pone.0006057.s001.tif]
