# Supplementary material for: Quantification of Normal Cell Fraction and Copy Number Neutral LOH in Clinical Lung Cancer Samples Using SNP Array Data
Source: PLoS One. 2009 Jun 26;4(6):e6057. doi: 10.1371/journal.pone.0006057 (PMC2699026; doi:10.1371/journal.pone.0006057)
Supplement: Table S1 — Size of the regions detected as CNNLOH using SOMATICS on Affymetrix and Illumina data from 9 Chronic Lymphocytic Leukemia samples described in Gunnarsson et al [18]. Ratio of the length of regions detected on Affymterix and Illimina data. Note that SOMATICS detects more CNNLOH using Affymetrix data than with Illumina data. (0.03 MB DOC) [file pone.0006057.s002.doc]

**Table S1. SOMATICS detects different regions of CNNLOH using data from the Affymetrix and Illumina platforms.**

| **Sample number** | **Length of CNNLOH regions**  **Affymetrix data (Mbp)** | **Length of CNNLOH regions**  **Illumina data**  **(Mbp)** | **Ratio of detected CNNLOH Affymetrix/**  **Illumina** |
| --- | --- | --- | --- |
| 1 | 96.6 | 51.3 | 1.9 |
| 2 | 168.5 | 8.9 | 19.2 |
| 3 | 213.2 | 14.3 | 14.9 |
| 4 | 127.0 | 9.6 | 13.2 |
| 5 | 126.1 | 39.5 | 3.2 |
| 7 | 330.3 | 51.2 | 6.5 |
| 8 | 485.4 | 44.0 | 11.0 |
| 9 | 368.6 | 10.2 | 36.3 |
| 10 | 253.2 | 14.4 | 17.5 |
