# Supplementary material for: Quantification of Normal Cell Fraction and Copy Number Neutral LOH in Clinical Lung Cancer Samples Using SNP Array Data
Source: PLoS One. 2009 Jun 26;4(6):e6057. doi: 10.1371/journal.pone.0006057 (PMC2699026; doi:10.1371/journal.pone.0006057)
Supplement: Text S1 — Choice of settings. (0.03 MB DOC) [file pone.0006057.s003.doc]

Text S1. Choice of settings:

The CNNLOH quantifier method uses a number of settings. Information regarding the choice of these settings is provided below:

**Window size**

A window of 140 consecutive SNP markers has been used for estimation of the proportion of tumor cells with CNNLOH. The size of the window is chosen to provide histograms of ABf values that can accurately determine the proportion of heterozygous cells. A larger experimental variation in ABf values from different loci will require a larger window to obtain equally accurate estimates. Optimal window size with high resolution of CNNLOH and accurate quantification of CNNLOH cells is expected to depend on the data. The user may want to vary the window size for their particular data and application.

**Number of simulated markers**

To estimate the average rate of heterozygosity genotypes on a large chromosomal region with no apparent genomic aberrations were used. In this case 1363 of 3712 marker loci used in this study were heterozygous. To simulate a sufficiently large number of loci to obtain sufficiently stable estimates of ABf histograms we chose to simulate 4 * 3712 =14848 loci and maintain the observed average rate of heterozygosity. The exact number of simulated loci is not important as long as it is large enough to provide sufficiently reproducible histograms.

**Distance measure for comparison of similarity between histograms**

As can be seen in Fig.2, the part of the histograms that is most affected by variation the proportion of heterozygous cells is in the middle of the histogram (around ABf=0.5). On the other hand uninformative markers have ABf values close to 0 or 1. In order to reduce the influence of the variation in the distribution of uninformative markers on the measure of similarity between histograms, we chose to use to use the Euclidean distance of the bins 5 to 16 that corresponds primarily to informative markers around ABf= 0.5.

**Criteria for determining the fraction of normal cells**

We used two criteria to be fulfilled in order to estimate the fraction of normal cells. There had to be deletions on at least two chromosomes that could be used to estimate C_2N_. The difference between the two lowest estimates should not exceed 5%. The criteria were chosen to allow normal variation in estimates of C_2N_ , but to exclude estimates with contribution from diploid tumor cells.
